# Nanomedicine and medicinal plants: Emerging symbiosis in managing lung diseases and associated infections

**DOI:** 10.17179/excli2022-5376

**Published:** 2022-10-27

**Authors:** Keshav Raj Paudel, Gabriele De Rubis, Nisha Panth, Sachin Kumar Singh, Dinesh Kumar Chellappan, Philip Michael Hansbro, Kamal Dua

**Affiliations:** 1 Centre of Inflammation, Centenary Institute and University of Technology Sydney; Faculty of Science, School of Life Sciences, University of Technology Sydney, Sydney 2007, Australia; 2 Discipline of Pharmacy, Graduate School of Health, University of Technology Sydney, Sydney, NSW 2007, Australia; 3 Faculty of Health, Australian Research Centre in Complementary & Integrative Medicine, University of Technology Sydney, 2007, Ultimo, Australia; 4 School of Pharmacy and Pharmaceutical Sciences, Lovely Professional University, Phagwara, Punjab 144411, India; 5 Department of Life Sciences, School of Pharmacy, International Medical University, Bukit Jalil 57000, Kuala Lumpur, Malaysia

## ⁯

Pulmonary diseases refer to a broad category of diseases (both acute and chronic) related to the lungs that include asthma, acute lung injury, chronic obstructive pulmonary disease (COPD), pulmonary hypertension, pulmonary fibrosis, and lung cancer. These diseases affect a significant percentage of the global population (Paudel et al., 2021[12]). Medicinal plants are gaining massive attention as promising therapeutics in alleviating respiratory diseases (Chan et al., 2021[2]; Prasher et al., 2020[15]). Advanced drug delivery systems have become well-established, with nanotechnology in particular, which has taken on new significance as an emerging field in the management of inflammatory lung diseases (Devkota et al., 2021[5]; Manandhar et al., 2022[8]).

Nanotechnology involves the formulation, characterization, and application of materials such as therapeutics and diagnostics that exist in the nanoscale range of 1 to 100 nm. As these nanostructures are in the size range or in the scale comparable to biological moieties, they may be engineered with nanotechnology for use in medical applications. The aim of nanomedicine is to utilize the physiochemical characteristics of potential nanomaterials in a way that favors diagnosis and treatment of various respiratory diseases (Kim et al., 2010[7]). In recent years, the concept of advanced therapeutic delivery systems using nanomedicine as a powerful tool in the management of lung diseases and associated infections has been rapidly progressing. The pathophysiological features of lung diseases, particularly airway remodeling and airway inflammation may be effectively targeted using novel drug delivery systems that generally include the active drug moiety loaded in nanocarriers such as dendrimers, lipid micelles, liposomes, polymeric nanoparticles, solid-lipid nanoparticles (SLN), and liquid crystalline nanoparticles (LCN) (Dhanjal et al., 2022[6]). These nanocarriers offer versatility in the management of lung diseases as observed through the findings from various *in vitro* experiments (example: human healthy and cancerous bronchoepithelial cell line, and macrophages) and *in vivo* experiments (example: urethane induced lung cancer bearing mice) (Chan et al., 2021[1]). Recent research trends have also highlighted the promising aspect of decoy oligonucleotides (such as NFκB) based nanomedicines in the management of lung diseases (Mehta et al., 2021[10]). In the section below, we have highlighted the protective role of medicinal plants/single compound-based nanomedicines identified from several *in vivo *and* in vitro* studies showcasing their mechanisms and promising aspects of nanomedicine in managing lung diseases.

Wang et al. (2013[20]) formulated curcumin-loaded solid lipid nanoparticles for the treatment of mice bearing human lung cancer (A549 cells) xenografts and compared their activity with plain/pure curcumin powder. Daily intraperitoneal injection of SLN-curcumin administered 5 days/week for 19 days at a dose of 200 mg/kg body weight resulted in the accumulation of curcumin both in the lung and in the tumor (detected by HPLC analysis). Interestingly, the inhibition of tumor growth by SLN-curcumin was 3.5-fold higher (69.3 %) compared to plain curcumin (19.5 %). The findings from the flow cytometry analysis and TUNEL staining (that analyzes DNA fragments during apoptosis) revealed that the reduction in tumor growth was because of apoptosis (but not necrosis) of tumor cells. In a similar way, the inhibition of tumor proliferation marker Ki67 was higher (33.9 %) with SLN-curcumin compared to the plain curcumin (20.0 %) (Wang et al., 2013[20]). On the other hand, chitosan encapsulation of curcumin was found to enhance the bioavailability and tissue retention of the formulation leading to an improved efficacy in preventing benzo[a]pyrene (a potent carcinogen) induced lung cancer in Swiss albino mice (Vijayakurup et al., 2019[18]). Apart from lung cancer, curcumin-SLN was also found to exert enhanced bioavailability and efficacy against allergic asthma induced by ovalbumin in a mice model by suppression of airway hyperresponsiveness, inflammatory cell infiltration, inhibition of cytokine expression particularly, IL-4 and IL-13 in bronchoalveolar lavage fluid (BALF) compared to the asthma (ovalbumin only) group. The anti-asthmatic activity of curcumin-SLN was superior to the plain/pure curcumin-treated group (Wang et al., 2012[21]).

A self-assembled polyjuglanin (a type of flavonoid found in walnut husks) nanoparticles loaded with chemotherapeutic agents such as doxorubicin and anti-Kras siRNA were formulated by Wen et al. (2017[22]) who investigated the efficacy of the nanoparticles to attenuate tumor growth in a BALB/c nude mice inoculated with lung cancer cell line; A569 and H69. The prepared nanoparticles significantly reduced tumor proliferation by inhibiting Ki-67 expression and thereby induced apoptosis of tumor cells. Comparatively, the anticancer activity of nanoparticles was better than free doxorubicin suggesting juglanin as a potent chemosensitizer (Wen et al., 2017[22]). 

Resveratrol-loaded lipid-core nanocapsules were designed by interfacial deposition of biodegradable polymers for oral delivery (5 mg/kg) to test their biological activity against acute lung injury model in A/J mice induced with lipopolysaccharide (LPS). Comparisons of efficacy of resveratrol-loaded nanocapsules were made with unloaded nanocapsules or free resveratrol. Interestingly, there was a notable reduction in LPS-induced leukocyte accumulation in the BALF, whereas the unloaded nanocapsules did not show any significant changes with leukocyte count compared to the LPS group. Only resveratrol-loaded but not unloaded nanocapsules or free resveratrol improved the impaired lung function (lung elastance) due to LPS treatment. In a similar way, the production of pro-inflammatory cytokines such as IL-6, KC, MIP-1α, MIP-2, MCP-1 and RANTES in lung tissue were inhibited significantly by resveratrol-loaded but not unloaded nanocapsules. Furthermore, only resveratrol-loaded nanocapsules reduced malondialdehyde (a marker of oxidative stress) and superoxide dismutase (an antioxidant enzyme that upregulates to compensate the oxidative stress due to LPS). LPS binds to the TLR4 receptor and provokes the activation of ERK and PI3K/Akt pathways leading to gene transcription and proinflammatory cytokine production. Only resveratrol-loaded nanocapsules reduced the protein expression of phosphorylated-ERK and phosphorylated-AKT (de Oliveira et al., 2019[4]).

Various phytochemicals have been designed using the nanotechnology approach to test their efficacy *in vitro* in various cell lines of respiratory systems. Naringenin is a bioflavonoid with diverse beneficial activity against chronic respiratory disease, however, its poor water solubility and bioavailability have limited its use (Chin et al., 2020[3]). Wadhwa et al. recently designed the LCNs of naringenin and studied the anti-inflammatory potential of the nanoformulation in LPS induced inflammation in human airway epithelium cells (BCi-NS1.1). The findings revealed that naringenin-loaded LCNs significantly reduced the expression of inflammatory genes; IL-6, IL-1β, IL-8 and TNF-α. The anti-inflammatory potency of naringenin-LCNs were similar to standard commercially available anti-inflammatory drug fluticasone propionate (Wadhwa et al., 2021[19]). Apart from their anti-inflammatory activity, phytochemical-loaded LCNs are also found to possess antioxidant activity in *in vitro *settings. A recent study has demonstrated the antioxidant activity of nanoparticles loaded with another bioflavonoid, rutin. Rutin-loaded LCNs in LPS-stimulated oxidative stress model were studied using a human bronchial epithelial cell line (BEAS-2B). The authors revealed that a low dose of rutin-LCNs (compared to a high dose of rutin pure compound in another similar study) drastically reduced the generation of reactive oxygen species and nitric oxide production that have further led to the inhibition of BEAS-2B apoptosis (Paudel et al., 2020[13]). The antioxidative activity of rutin-loaded LCNs was due to the upregulation of antioxidant genes Gclc and Nqo-1 and the downregulation of Nox2B and Nox4 (Mehta et al., 2021[11]). Likewise, rutin-loaded LCNs showed potent anti-cancer activity against the lung cancer cell line A549 by inhibiting cell proliferation, migration, colony formation and by inducing apoptosis of A549 cells (Paudel et al., 2021[14]). The anticancer activity of another potent plant alkaloid, berberine, when loaded in LCNs, was revealed to be due to the inhibition of CXCL-8, CCL-20 and HO-1 gene expressions that play an important role in cancer cell proliferation and migration (Mehta et al., 2021[9]). Apart from LCNs, chitosan nanoparticles are excellent carriers of active drug moieties. In this aspect, boswellic acid-loaded chitosan nanoparticles have reportedly shown remarkable cytotoxicity in A549 cells. In comparison to the IC_50_ value of 29.59 μM for free drug (boswellic acid), the IC_50_ value of boswellic acid loaded chitosan nanoparticles was 17.29 μM which explains the potency of nanoparticles over free drug (Solanki et al., 2020[17]). 

Curcumin was loaded into polyvinylpyrrolidone nanoparticles by Yen et al. (2013[23]) to study its beneficial role in TNF-α-treated lung epithelial cells. The overexpression of ICAM-1, reactive oxygen species production and cell adhesion were reported to be ameliorated by curcumin nanoparticles. Mechanistically, the antioxidant activity of curcumin nanoparticles was due to the inhibition of p47 (phox) and MAPKs/AP-1 pathways (Yen et al., 2013[23]). *Pseudomonas aeruginosa* and *Staphylococcus aureus* are common pathogens that attack lungs resulting in pneumonia and other respiratory complications. A hybrid ofloxacin and eugenol (also referred as clove oil) co-loaded chitosan-SLNs were formulated by Rodenak-Kladniew et al. (2019[16]) that were found to possess enhanced antimicrobial activity against *P. aeruginosa *and* S. aureus*. While the free and nano-encapsulated ofloxacin showed minimum inhibitory concentration (MIC) below 1.0 µg/ml, the MIC values shown by ofloxacin and eugenol co-loaded chitosan-SLN were 6.1- to 16.1-folds less than free and nano-encapsulated ofloxacin. When the nanoparticles were tagged with a fluorescent label, the ability of nanoparticles to interact with the bacterial cell membrane was observed suggesting potent antimicrobial activity of the clove oil (Rodenak-Kladniew et al., 2019[16]).

Although plenty of clinical trials involving phytochemicals have been conducted and investigated for their potential to be used in clinical practice for the management of chronic respiratory diseases, the number of clinical trials involving phytochemicals designed with a nanotechnology approach is very limited. Considering the immense potential of phytochemicals, there is an excellent platform for researchers in the field of nanomedicine/nanotechnology to test such candidate drugs by formulating them into various forms of nanomedicine with enhanced physiochemical properties to generate favorable biological activity against lung disease and associated infections.

The various highlighted recent case studies demonstrate and support the amalgamation of nanotechnology with phytochemicals. The nanoscale encapsulation of therapeutic moieties obtained from medicinal plants has proven to be beneficial with the help of nanocarrier-based systems that have provided a new direction to pulmonary clinics.

## Notes

Philip Michael Hansbro and Kamal Dua (Discipline of Pharmacy, Graduate School of Health, University of Technology Sydney, Sydney, NSW 2007, Australia; E-mail: Kamal.Dua@uts.edu.au) contributed equally as corresponding author.
